# Molecular and Serological Detection of *Toxoplasma gondii* in Two Species of Rodents: *Ctenodactylus gundi* (Rodentia, Ctenodactylidae) and *Psammomys obesus* (Rodentia, Muridae) From South Tunisia

**DOI:** 10.1002/vms3.70371

**Published:** 2025-04-28

**Authors:** Faten Bouaicha, Safa Amairia, Yosra Amdouni, Khawla Elati, Boubaker Bensmida, Mourad Rekik, Mohamed Gharbi

**Affiliations:** ^1^ Laboratory of Parasitology, National School of Veterinary Medicine of Sidi Thabet University of Manouba Sidi Thabet Tunisia; ^2^ Centre Hospitalier Universitaire Vétérinaire–Animaux de production, École Nationale Vétérinaire d'Alfort Maisons Alfort France; ^3^ Institute of Parasitology, Vetsuisse Faculty University of Bern Bern Switzerland; ^4^ Institute for Parasitology and Tropical Veterinary Medicine, Freie Universität Berlin Berlin Germany; ^5^ Commissariat Régional de Développement Agricole de Tataouine Tataouine Tunisia; ^6^ International Centre for Agricultural Research in the Dry Areas (ICARDA) Amman Jordan

**Keywords:** ELISA, PCR, rodents, Sahara, Toxoplasma gondii, Tunisia

## Abstract

The molecular and serological prevalence of *Toxoplasma gondii* infection was investigated among rodents living in desertic areas in the Tataouine district, in the south of Tunisia. A total number of 43 rodents were captured from four sites classified as arid and Saharan climatic zones. Sera, hearts, spleens and brains were collected from each rodent. Sera were tested for the presence of anti‐*T. gondii* IgG by the ELISA technique. PCR was used to detect *T. gondii* DNA from different tissues. Two rodent species were identified as *Ctenodactylus gundi* (Rodentia, Ctenodactylidae) (*N* = 28; 65%) and *Psammomys obesus* (Rodentia, Muridae) (*N* = 15; 35%). The overall molecular prevalence of *T. gondii* was 39% (17/43). Infection prevalences were higher in *C. gundi* (53.6%; 15/28) compared to *P. obesus* (13.3%; 2/15). In both species, the brain was the most infected organ (*p* = 0.02). No significant difference was recorded for the two rodent species according to gender and sampling sites (*p* > 0.05). The overall seroprevalence was up to 34.9% (15/43). It was higher in *C. gundi* (43%; 12/28) compared to *P. obesus* (20; 3/15) (*p* = 0.02). These results highlight a high infection level of *T. gondii* in desertic rodents. More investigations are required to understand the role of other desertic mammals and to identify the genotype circulating in the Tunisian Sahara.

## Graphical abstract

Rodents play a critical role in the life cycle of *Toxoplasma gondii* as intermediate hosts. Molecular and serological results reveal a high infection rate in two species of desert rodents.

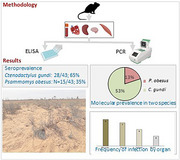

## 1 Introduction

*Toxoplasma gondii* is an intracellular protozoan parasite that infects a wide variety of warm‐blooded organisms, including mammals and birds. Toxoplasmosis, caused by *T. gondii*, is a highly prevalent zoonosis that infects approximately one‐third of the world's human population (Tenter et al. 2000). The life cycle of the obligate parasite involves the felids as a definitive host where the sexual reproduction occurs and an intermediate host which involves a large variety of animals (Dubey 2020). Rodents are essential intermediate hosts for *T. gondii* as they are preyed upon by cats and other felids. Consequently, they serve as the primary source of infection for members of the Felidae family, facilitating the establishment of the parasite life cycle (Dubey et al. 2021).

Rodents have been demonstrated to harbour infectious *T. gondii* (Hejlíček et al. 1997), and they become infected after ingesting soil, vegetation, or water contaminated with oocysts or eating infected animal tissues containing *T. gondii* cysts (Dabritz et al. 2008). Among mammals, rodents represent the most diversified and largest order, representing approximately 42% of mammalian biodiversity (Galeh et al. 2022). Along with toxoplasmosis, several zoonotic infections are carried out by rodents that act mainly as reservoirs (Taylor et al. 2008) and play an important role in their transmission and dissemination.

In Tunisia, a total of 27 rodent species have been identified, belonging to 12 genera. The Muridae family, characterized by its diversity, comprises 21 species present in Tunisia (Selmi et al. 2021). Such extensive diversity could represent a significant risk factor for the transmission and spread of zoonotic pathogens. Many wild rodent species have been incriminated in the transmission of various microorganisms to both animals and humans, such as *Psammomys obesus*. It is a desert fat sand rat usually used as a model to study diabetes (King and Austin 2017) and is a reservoir of *Leishmania* in Asia, Africa and the Middle East (Echchakery et al. 2015). In Tunisia, this rodent species was found to carry *Bartonella*, *Borrelia*, and *Babesia* (Fichet‐Calvet et al. 2000).

*Ctenodactylus gundi* is a widespread small rodent living in desert and semi‐desert rocky areas of North Africa (Ghawar et al. 2018). This species was initially used for leishmaniosis diagnosis, and a new parasite was discovered in these rodents, leading to the naming of *T. gondii*. At that time, *T. gondii* was first discovered in 1908 in Tunisia, precisely in the Pasteur Institute of Tunis, by Nicolle and Manceaux during experiments on leishmaniosis (Despommier et al. 1995).

Subsequently, several studies were conducted, revealing the role of the *C. gundi* species as a potential reservoir of leishmaniosis in Tunisia (Bousslimi et al. 2012; Ghawar et al. 2018).

Although *C. gundi* was recognized as a natural reservoir of *T. gondii*, little is known about its potential role in disseminating these pathogens to domestic animals and their environment.

Molecular protocols for *T. gondii* detection have been optimized, and several markers, such as the *B1* gene, have been frequently used due to their specificity and sensitivity (Burg et al. 1989; Liesenfeld et al. 1994; Reischl et al. 2003). Some other studies have used ITS‐1 and 18S rDNA fragments for *T. gondii* detection, with sensitivity similar to that of the *B1* gene (Hurtado et al. 2001; Calderaro et al. 2006). The 529 bp repetitive element, identified by Homan et al. (2000), is highly sensitive for detecting *T. gondii*, but some strains lack or have mutations in this fragment (Reischl et al. 2003). Recent studies also indicate that the number of copies of the 529 bp sequence in the *T. gondii* genome is lower than previously thought, which raises concerns about its reliability for quantification in clinical diagnostics (Costa and Bretagne 2012).

Several studies reported high *T. gondii* prevalence in both humans and domestic animals (Rouatbi et al. 2019), yet the *T. gondii* infection in this wild rodent in the country remains unknown. In this context, the objective of the current study was to estimate the *T. gondii* infection rate in desert rodents within the Tataouine district of southern Tunisia. This contributes to a better knowledge of desert rodents’ role in the epidemiology of *T. gondii* infection.

## 2 Materials and Methods

### 2.1 Rodent Trapping and Identification

**FIGURE 1:**
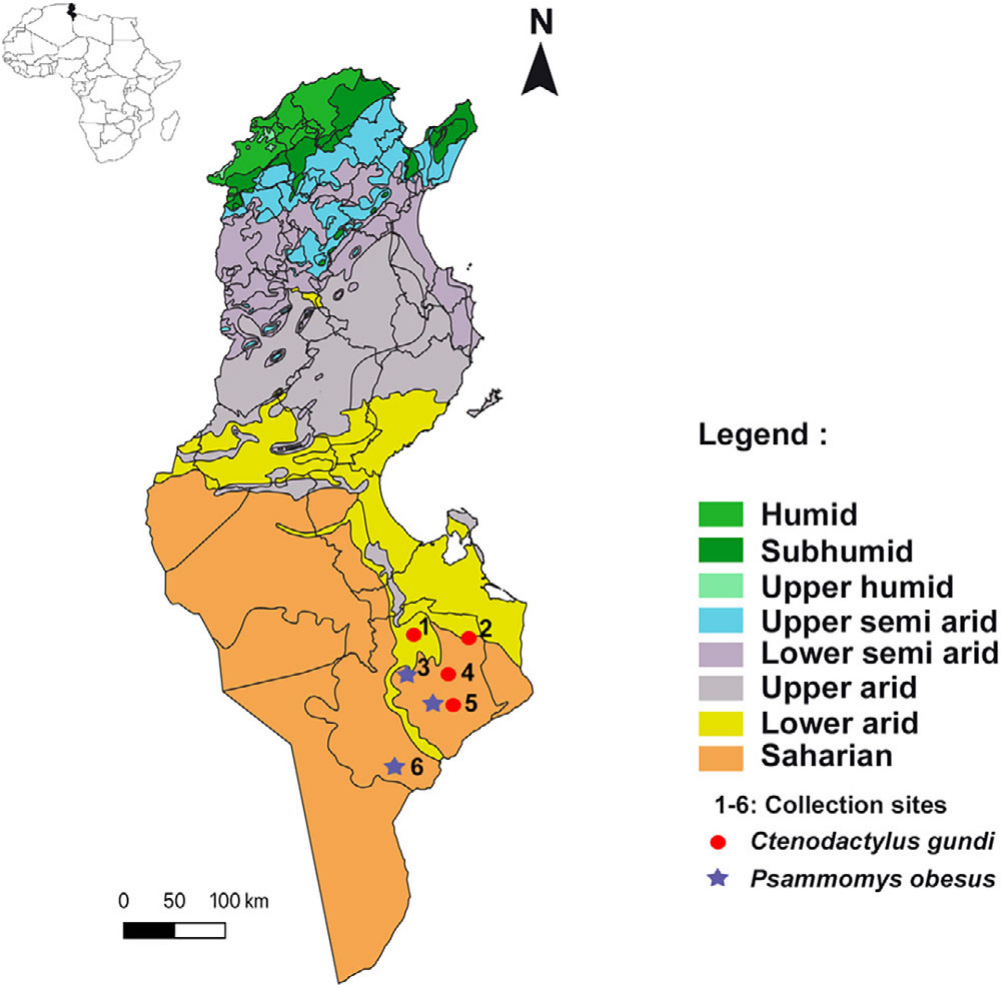
Map of Tunisia showing the sampling sites of rodents (QGIS).

The present study was carried out in the Tataouine district (10°27′E, 32°55′N) in two bioclimatic zones: arid and Saharan. With a total area of 38,889 km^2^, Tataouine is the largest district in Tunisia. Between January and August 2021, Sherman live traps were placed in six sites, 30 km apart each (Figure 1). The choice of sampling sites was based on farmers’ declaration of rodents’ presence. Olive, date and barley grains were put in each trap as bait. All the traps were controlled every 2 days for the presence of rodents for 6 months. A total of 43 rodents were collected alive and transported to the laboratory, where their weight and sex were determined. Morphological identification to the species level was performed according to the key of Cunningham and Moors (1996).

The study protocol was read and approved by the ethics committee of the National School of Veterinary Medicine of Sidi Thabet, registered under the number 0123/2020 ENMV.

### 2.2 Rodent Autopsy and Organ Sampling

Live‐trapped rodents were anaesthetized with ether and euthanized with pentobarbital (100 mg/kg injected intraperitoneally). Immediately after euthanasia, blood samples in dry tubes were collected from the jugular vein. Sera were collected and conserved at −20°C in Eppendorf tubes after blood centrifugation. Animal corpses were then dissected according to the protocol of Treuting and Snyder (2015). The heart, quadriceps muscle and spleen were collected from all captured rodents. Brain samples were collected from 14 rodents only. All the samples were aseptically collected and then conserved at −20°C until used.

### 2.3 DNA Extraction and Universal Polymerase Chain Reaction

The genomic DNA extraction was carried out from the spleen, brain, heart and muscle tissue samples of each rodent. The extraction step was preceded by a homogenization of the tissue samples in liquid nitrogen. Briefly, 30 mg of each organ was washed with 1 mL of sterile distilled water, mixed with nuclei lysis solution and followed by three cycles of freezing (at −20°C) and thawing (at +50°C) of 5 min each. Then, 10 µL of proteinase K was added to the mixture, and the samples were incubated overnight at 37°C. The DNA extraction was performed using the Wizard Genomics DNA extraction kit (Promega, Madison, Wisconsin, USA) according to the manufacturer's instructions and then stored at −20°C until used.

To check the quality of DNA, a conventional PCR was performed using a set of universal primers (forward: 5ʹ‐AACCTGGTTGATCCTGCCAGT‐3ʹ and reverse: 5ʹ‐GGCACCAGACTTGCCCTC‐3ʹ), coding for the 18S rRNA gene (Mikhail et al. 2017). PCR reactions were carried out using a master mix consisting of 2.5 µL of PCR buffer, 2 µL of MgCl_2_ (25 mM), 1.5 µL of each primer (10 µM), 0.5 µL of dNTP mix (10 mM), 0.25 µL of Taq polymerase (2 U, Vivantis, Chino, California), 3 µL of DNA template and completed to 25 µL with sterile distilled water. The universal PCR was carried out in a thermocycler under the following conditions: initial denaturation for 5 min at 94°C, followed by 25 cycles (94°C, 59°C and 72°C for 50 s each) and a final extension for 10 min at 72°C.

### 2.4 PCR Amplification of *T. gondii*

Positive DNA samples from universal PCR were subject to specific nested PCR to amplify a 227‐bp fragment of the ITS1 gene of *T. gondii* as described by Hurtado et al. (2001). Two successive amplifications were performed using two primer pairs. The specific primers NN1 (5ʹ‐TCAACCTTTGAATCCCAA‐3ʹ) and NN2 (5ʹ‐CGAGCCAAGACATCCATT‐3ʹ) were used as internal primers for primary PCR that amplifies a 340 bp region of ITS1 common to *T. gondii* and *Neospora caninum*. The second primer set, Tg‐NP1 (5ʹ‐AACGGGCGAGTAGCACCTGAGGAGA‐3ʹ) and Tg‐NP2 (5ʹ‐TGGGTCTACGTCGATGGCATGACAAC‐3ʹ), were used for secondary PCR as external primers to detect a specific region of *T. gondii*. Both PCR reactions contained 2.5 µL of PCR buffer, 0.25 µL of each external primer and 1 µL of each internal primer (10 µM), 0.5 µL of each dNTP mix (10 mM), 1 µL of MgCl_2_ (50 mM), 0.5 µL of *Taq* polymerase and 2 µL of each DNA sample in a total reaction volume of 25 µL.

The DNA amplification was performed using the following cycling program: initial denaturation for 5 min at 95°C, followed by 35 cycles (denaturation at 95°C for 1 min, annealing at 55°C for 1 min and elongation at 72°C for 5 min) and a final extension at 72°C for 5 min.

Positive control (purified *T. gondii* DNA) and negative controls (water DEPC‐treated) were included in each PCR run. All PCR products were analysed using 2% agarose gel electrophoresis in TBE buffer containing 0.05% ethidium bromide. DNA bands were visualized under ultraviolet light.

### 2.5 Serological Analyses

The sera were tested for the presence of anti‐*T. gondii* IgG antibodies using a commercial species‐independent Enzyme‐Linked Immunosorbent Assay (ELISA) ID screen *Toxoplasmosis* Indirect Multispecies (IDvet, Montpellier, France) according to the manufacturer's recommendations. *T. gondii* P30 antigen and anti‐IgG‐HRP were used as a coated antigen and a conjugate, respectively. Optical densities were measured with an ELISA plate reader set at 450 nm wavelength. After plate validation, the serum was considered positive if the following ratio was higher than 30%: 100× optical density (OD) of the sample/OD of positive control serum.

### 2.6 Statistical Analyses

Exact confidence intervals for prevalence rates were calculated at the 95% level (95% CI). A comparison of the difference in serological and molecular prevalence according to sampling sites, rodent species and different infected organs was performed using the chi‐square Mantel–Haenszel test (Cohen et al. 1960).

## 3 Results

### 3.1 Rodents’ Population

The 43 trapped rodents belonged to two species, namely, *C. gundi* (Rodentia, Ctenodactylidae) (*N* = 28; 65%) and *Psammomys obesus* (Rodentia, Muridae) (*N* = 15; 35%). The sex ratio (M:F) was 1.6 (27/16). *C. gundi* was collected from four sites classified as arid (10/28) and Saharan climatic zones (18/28), respectively. Six *P. obesus* rodents were collected from the arid bioclimatic zone and nine from the Saharan bioclimatic zone.

### 3.2 Serological Findings

Out of 43 tested rodent sera, 15 were positive for anti‐*T. gondii* antibodies, resulting in a seroprevalence of 34.9% (15/43; 95% CI, 20.6–49.1). The seroprevalence was significantly higher in *C. gundi* at 42.8% (12/28; 95% CI, 24.5–61.2) compared to *P. obesus* at 20% (3/15; 95% CI, 0–40.2) (*p* = 0.02) (Table 1). *T. gondii* infection seroprevalence tends to be higher in *C. gundi* females than *P. obesus* ones (*p* = 0.08). Similar trends were observed for *C. gundi* samples collected from the Saharan zone, which were more seropositive than *P. obesus*, collected from the same zone. These results were statistically not significant (*p* = 0.30) (Table 1).

**TABLE 1:** Serological prevalence of *Toxoplasma gondii* in wild rodents in the Tataouine district (Southern Tunisia) according to animal species, gender and sampling sites.

		*Ctenodactylus gundi*	*Psammomys obesus*	
Items		Positive/examined (prevalence in %) 95% CI	Positive/examined (prevalence in %) 95% CI	*p* value
Bioclimatic zone	Arid	4/10 (40) [9.6–70.3]	2/6 (33.3) [0–71]	0.30
	Saharan	8/18 (44.4) [21.5–67.4]	1/9 (11.1) [0–31.6]	
Gender	Male	8/18 (44.4) [21.5–67.4]	3/9 (33.3) [2.5–64.1]	0.08
	Female	4/10 (40) [9.6–70.3]	0/6 NA	
**Total**		12/28 (42.8) [24.5–61.2]	3/15 (20) [0–40.2]	0.02^*^

Abbreviations: CI, confidence interval; NA, not applicable.

* Statistically significant (*p* ≤ 0.05).

### 3.3 Molecular Findings

#### 3.3.1 *T. gondii* Infection Prevalence in Rodents

Among the 43 studied rodents, 39% (17/43; 95% CI, 25–54.1) had at least one infected organ by *T. gondii*. The molecular infection prevalence was significantly higher in *C. gundi* at 53.5% (15/28; 95% CI, 35–72) compared to *P. obesus* at 13.3% (2/15; 95% CI, 0–30.5) (*p* = 0.03). The molecular infection prevalence of *T. gondii* was significantly higher in *C. gundi* females (70%) (7/10; 95% CI, 41.6–98) compared to *P. obesus* females (16.7%) (1/6; 95% CI, 0–46.5) (*p* = 0.045) (Table 2). In the same trend, when considering the Saharan bioclimatic zone, the molecular infection prevalence was significantly higher in *C. gundi* compared to *P. obesus* (55.5%) (10/18; 95% CI, 32.6–78.5) and (11.1%) (1/9; 95% CI, 0–31.6), respectively (*p* = 0.02). However, no significant difference in infection prevalence between rodents from arid bioclimatic zones was recorded (*p* = 0.3) (Table 2).

**TABLE 2:** Molecular prevalence of *Toxoplasma gondii* infection in wild rodents in the Tataouine district (Southern Tunisia) according to animal species, gender and sampling sites.

		*Ctenodactylus gundi*	*Psammomys obesus*	
Items		Positive/examined (prevalence in %) 95% CI	Positive/examined (prevalence in %) 95% CI	*p* value
Bioclimatic zone	Arid	5/10 (50) [19–81]	1/6 (16.7) [0–46.5]	0.02
	Saharan	10/18 (55.6) [32.6–78.5]	1/9 (11.1) [0–31.6]	
Gender	Male	8/18 (44.4) [21.5–67.4]	1/9 (11.1) [0–31.6]	0.045
	Female	7/10 (70) [41.6–98.4]	1/6 (16.7) [0–46.5]	
**Total**		15/28 (53.6) [35.1–72]	2/15 (13.3) [0–30.5]	0.03^*^

Abbreviations: CI, confidence interval; NA, not applicable.

* Statistically significant (*p* ≤ 0.05).

#### 3.3.2 Prevalence of *T. gondii* Infection in Rodents’ Organs

**FIGURE 2:**
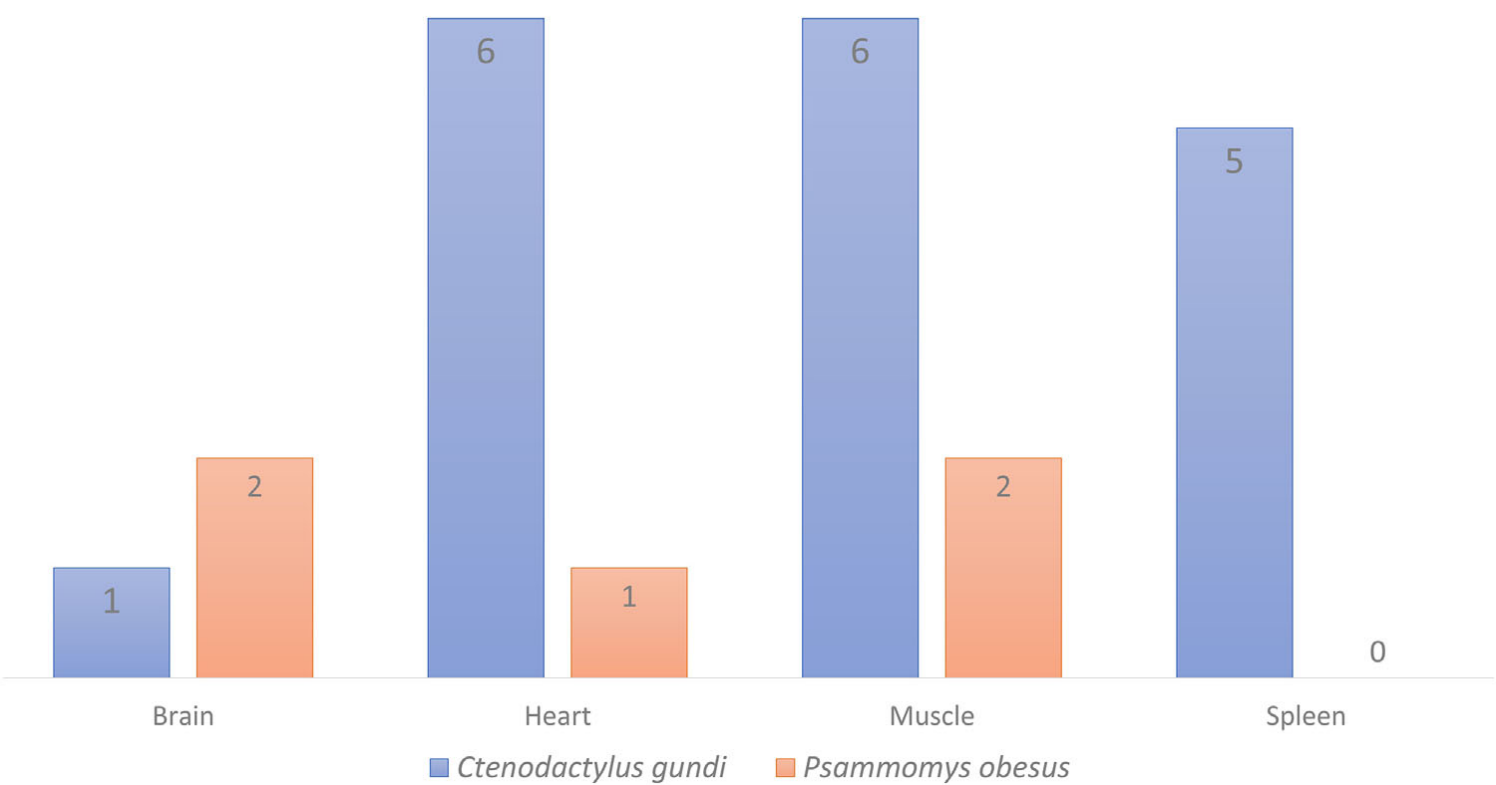
Frequency of infected rodent organs by *Toxoplasma gondii* in Tataouine district (Southern Tunisia).

In both rodent species, the molecular infection prevalence varied significantly according to the organ. The molecular positivity frequencies are 21.4% (3/14; 95% CI, 0–43) in the brains, 18% (8/43; 95% CI, 6.9–30) in the muscles, 16% (7/43; 95% CI, 5.2–27.3) in the hearts, and 11% (5/43; 95% CI, 2–21.2) in the spleens (*p* = 0.02) (Figure 2).

In *C. gundi* infection prevalence varied significantly across different tissues (*p* = 0.03). The prevalence was 20% in the brain (1/5; 95% CI, 0–55), 21% in both the heart (6/28; 95% CI, 6.2–36.6) and muscle (6/28; 95% CI, 6.2–36.6), and 18% in the spleen (5/28; 95% CI, 3.6–32).

However, no significant difference was found when testing *P. obesus*’ muscle (13%) (2/15; 95% CI, 0–30.5), brain (11%) (1/9; 95% CI, 0–31.6), heart (6.6%) (1/15; 95% CI, 0–19.3) and spleen (0) samples for the presence of *T. gondii* DNA (*p* = 0.13) (Figure 2). There was also no difference when comparing *T. gondii* infections for the two rodents’ species, according to the sampling region (*p* > 0.05).

#### 3.3.3 Frequency of Infected Rodents’ Organs by *T. gondii*

Among the 17 infected rodents, 58.8% (10/17; 95% CI, 42–75) had only one infected organ, 23.5% had two infected organs (4/17; 95% CI, 13%–33%), 11.7% had four infected organs (2/17; 95% CI, 4%–19%) and 5.8% had three infected organs (1/17; 95% CI, 0.7%–10%).

## 4 Discussion

In the present study, we examined 43 desertic rodents from south Tunisia in order to estimate the molecular and serological *T. gondii* infection prevalence in rodents collected in the Tataouine district, Southern Tunisia.

The low number of collected specimens may be due to the short time of capture and the difficulty of accessing the desertic trapping zones. Thus, the captured species doesn't reflect the biodiversity and biomass of rodents in the Tunisian desert. Still, *C. gundi* and *Psammomys obesus* represent the two major species in the Tunisian Sahara.

The distribution of collected specimens can be explained by the biological characteristics of each rodent species. In fact, *C. gundi*, also known as the North African gundi, inhabits crevices within the rocky mountainous areas surrounding the villages (Meddour et al. 2022). As gregarious diurnal mammals, gundis are easy prey for domestic and wild felids. As definitive hosts, numerous wild felid species are present in the Tunisian desert, including *Felis libyca*, *Felis margarita*, Caracal, *Leptailurus serval* and *Acinonyx jubatus*. Their role in maintaining the *T. gondii* cycle as a predator of rodent species is highly probable but remains unstudied, including aspects such as their geographic distribution and the diversity of *T. gondii* strains they harbour (Jeljli et al. 2024; Lachkhem et al. 2021). Domestic cats are also present near human settlements and livestock, creating an interface where *T. gondii* transmission between wild and domestic hosts can occur. Moreover, gundis are also hunted by local inhabitants for their consumption (Ghawar et al. 2018), which may represent a pattern of human contamination. *Psammomys obesus*, the fat sand rat, is present in the North African countries from Mauritania to Egypt. Its local distribution is governed by the disponibility of the halophytic Chenopodiaceae plant species on which they feed (Karmaoui et al. 2022; Pipano et al. 2002).

As in other North African countries, in Tunisia, *P. obesus* and *C. gundi* are reservoirs of *Leishmania* parasites causing cutaneous leishmaniosis in Tunisia and represent a significant source of epidemics (Aoun and Bouratbine 2014; Ghawar et al. 2018). To the best of our knowledge, the current status of *T. gondii* infection in wild and periurban rodents in Tunisia is unknown.

In Tunisia, several serological surveys were conducted on livestock animals that revealed different seroprevalences of *T. gondii* infection of 31.2% (24/77), and 17.7% (28/158) in goats and horses, respectively (Amairia et al. 2016; Boughattas et al. 2011). However, there are no studies on *T. gondii* infection prevalence in rodents. The present study revealed a high seroprevalence of *T. gondii* infection in Saharan rodents, 35% (15/43). These results were not expected since the reported values in other studies were lower: 3.8% (3/79) in Egypt and 3.6% (44/1205) in Senegal in urban rodents (Mikhail et al. 2017; Brouat et al. 2018).

Moreover, a systemic meta‐analysis that assessed *T. gondii* seroprevalence in rodents between 1970 and 2018, reported a mean seroprevalence of anti‐*Toxoplasma* IgG antibodies in rodents of 6% with a variation between countries. Using the modified agglutination test (MAT), the lowest and highest prevalence rates were reported in Norway (0.1%; 1/732) and Nigeria (100%; 104/104), respectively (Galeh et al. 2020).

The variation of seroprevalence observed in the different studies may be explained by several factors: (i) the abundance and density of definitive hosts in the study zone, (ii) the difference of abiotic factors that affect the sporulation of *Toxoplasma* oocysts and (iii) the performance of the used methods.

The high seroprevalence in our study is due to the local environment around captured rodents. Indeed, humid and warm climates are favourable conditions for the survival of oocysts. This microclimate can be found in the crevices within the Rocky Mountains or near camel herds. In addition, fossorial species, such *as C. gundi*, are frequently in contact with soil and may eat earthworms, mechanical carriers of *T. gondii* (Afonso et al. 2007).

Our results revealed for the first time a high rate of molecular infection in wild desertic rodents, 39% (17/43). *T. gondii* DNA was detected in both *C. gundi* (53%; 15/28) and *P. obesus* (6.5%; 2/15), with a greater positivity of *C. gundi* compared to *P. obesus*. This result remains inconclusive and cannot confirm the sensibility of one species compared to another. More studies are needed to understand the role of each rodent species in the life cycle of *T. gondii*.

In Tunisia, several studies were conducted to estimate the molecular prevalence of *T. gondii* infection in animals. Amdouni et al. (2017) estimated the overall molecular *T. gondii* prevalence in sheep, goats and cattle to be 33.3% (50/150), 32.5% (39/120) and 19.3% (29/150), respectively.

More recently, an investigation of *T. gondii* infection in free‐range chicken meat and offal (heart and gizzard) from north Tunisia revealed a global molecular prevalence of 78.3% (47/60) by targeting the ITS‐1 region (Zrelli et al. 2022). It is important to note that no studies have been conducted in the Tataouine district to assess the prevalence of *T. gondii* infection in different animal species.

Most of the studies on the role of rodents in toxoplasmosis transmission focused on peri‐urban rodents, especially the brown rat (*Rattus norvegicus*) and the house mouse (*Mus musculus*). Both species live near humans and share their habitats with domestic felids (Aviat et al. 2009; Runge et al. 2013). This close proximity explains the contribution of peri‐urban rodents in the life cycle of *T. gondii* (Kalmár et al. 2023). However, the role of wild species is less documented. Epidemiological surveys conducted in several countries reported a molecular infection rate by *T. gondii* in wild and urban rodents (black rats: *Rattus*, brown rat: *Rattus norvegicus*, and the house mouse, *Mus musculus*) varying between 7.9% and 15%, using nested PCR (Afonso et al. 2007; Krijger et al. 2020; Zou et al. 2022).

In our study, *T. gondii* DNA was detected in all tested organs: muscle (18.6%; 8/43), spleen (11.6%; 5/43), heart (16%; 7/43) and brain (21%; 3/14). A previous study reported a molecular prevalence of *T. gondii* infection in the apex heart of sheep that ranged between 5.7 and 25.5% (Gharbi et al. 2013). This result suggests that rodents, as intermediary hosts, may play a role as important as sheep in the transmission of toxoplasmosis in Saharan regions.

In Tunisia, several studies describing the genetic diversity of *T. gondii* strains among domestic animals, mainly free‐ranging chickens and sheep, are conducted. The results reveal a diverse genetic landscape, with a significant presence of Type II strains followed by Type III (Lachkhem et al. 2021) and the emergence of atypical lineages (Boughattas et al. 2011, Lahmar et al. 2020). This genetic variability has important implications for understanding the epidemiology and pathogenicity of toxoplasmosis in the region.

Our results show a high level of *Toxoplasma* infection in Saharan rodents collected in the Tataouine district. The proximity of rodents and *Toxoplasma* reservoirs suggests that *Toxoplasm*a‐infected rodents may represent a public health risk in this area.

In the Sahara, gundis (*C. gundi*) could be captured and consumed by some locals. This practice increases the risk of human contamination, especially when meat is undercooked.

## 5 Conclusion

Our results confirm that *T. gondii* is prevalent in at least two rodent species *(C. gundi* and *P. obesus*) in the Tataouine district (South Tunisia), where they live, constituting prey for felids. These results contribute to a better knowledge of *T. gondii* epidemiology by extending the list of intermediate animal species in Tunisia. Also, they highlight the importance of wild rodents in spreading the infection in both humans and animals. However, more research works are required to evaluate the status of other sympatric micromammals. It is also interesting to proceed to a phylogenetic characterization of *T. gondii* circulating among wild animals to compare them with *T. gondii* strains circulating in both humans and domestic animals.

## Author Contributions

**Faten Bouaicha**: investigation, methodology, data analyses, writing – original draft. **Safa Amairia**: investigation – writing. **Yosra Amdouni**: investigation. **Khawla ELATI**: writing. **Boubaker Bensmida**: investigation. **Mourad Rekik**: funding acquisition. **Mohamed Gharbi**: conceptualization, supervision, funding acquisition, writing – review and editing.

## Ethics Statement

The study protocol was read and approved by the ethics committee of the National School of Veterinary Medicine of Sidi Thabet, registered under the number of 0123/2020 ENMV. Rodent trapping, handling and the experimental protocol presented in this study comply with the current available international guidelines of animal welfare in the care and use of animals in research.

## Conflicts of Interest

The authors declare no conflicts of interest.

### Peer Review

The peer review history for this article is available at https://www.webofscience.com/api/gateway/wos/peer‐review/10.1002/vms3.70371.

## Data Availability

The data that support the findings of this study are available from the corresponding author upon reasonable request.
